# An initial *G* value of hydrated electrons updated by a dynamic Monte Carlo simulation

**DOI:** 10.1039/d6ra00147e

**Published:** 2026-03-13

**Authors:** Takeshi Kai, Tomohiro Toigawa, Yusuke Matsuya, Yuho Hirata, Hidetsugu Tsuchida, Akinari Yokoya

**Affiliations:** a Nuclear Science and Engineering Center, Japan Atomic Energy Agency 2-4 Shirane Shirakata, Tokai-mura, Naka-gun Ibaraki 319-1195 Japan; b Faculty of Health Sciences, Hokkaido University Kita-12 Nishi-5, Kita-ku Sapporo Hokkaido 060-0812 Japan; c Department of Nuclear Engineering, Kyoto University Nishikyo-ku Kyoto 615-8530 Japan; d Quantum Science and Engineering Center, Kyoto University Gokasho Uji Kyoto 611-0011 Japan; e Institute for Quantum Life Science, National Institutes for Quantum Science and Technology 4-9-1 Anagawa, Inage-ku Chiba-shi 263-8555 Japan

## Abstract

In the radiolysis of water vapour, we can easily categorise ionisation and electronic excitation; however, the ratio of ionisation and electronic excitation for liquid water remains uncertain. The ratio is intrinsically related to the kind of radiolytic species generated. The complexity of subsequently induced DNA damage in a living cell exposed to radiation depends on the type of radiolytic species generated. To address this critical issue, we estimate the ratio of ionisation and electronic excitation from delocalised and localised components of secondary electrons respectively, using time-dependent simulation methods based on a Monte Carlo code and molecular dynamics. We also investigate the primary electron energy dependence of the ionisation (*i.e.*, initial hydrated electron) yields after irradiation with 20 eV–30 keV electrons in liquid water. The estimated yields at 1 ps above 1 keV agree well with previous data in the literature, whilst those below 1 keV differ markedly from some conventional simulations. Generally, initial yields of hydrated electrons depend on the type of cross sections and branching ratios modelled, whilst our code provides initial yields based on femtosecond dynamics Monte Carlo simulations of secondary electrons, and will contribute significantly to various research fields involving water radiolysis.

## Abstract 2

Unlike conventional codes, our code provides initial yields of hydrated electrons based on femtosecond dynamics Monte Carlo simulations of secondary electrons and will contribute to various research fields involving water radiolysis.

## Graphical abstract

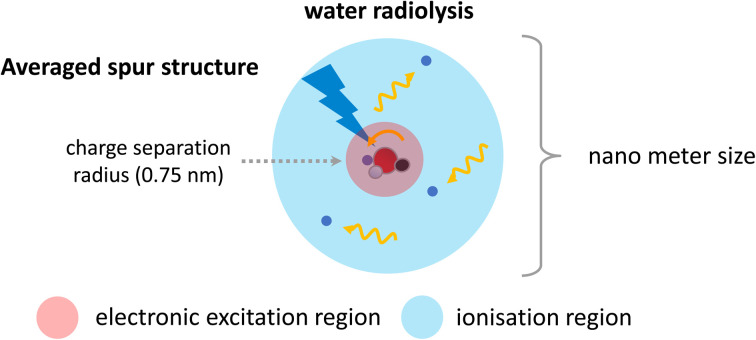

## Introduction

When high-energy electrons are incident to water, δ-rays with an energy of ∼10 keV and Auger electrons with an energy of ∼500 eV are occasionally produced; most of the produced electrons are low-energy secondary electrons with an energy of ∼10 eV. Such secondary electrons delocalise from the parent atom in the order of femtoseconds and hydration proceeds.^1–6^ After a few 10 ps, hydration is completed and many isolated spurs are distributed heterogeneously along the electron track. The spurs contain several radiolytic species such as hydrated electrons (e_aq_^−^), hydroxyl radicals (˙OH), and hydronium cations (H_3_O^+^), in which intra-spur chemical reactions begin after ∼100 ps.^7^ Inter-spur reactions proceed after a few hundred ns, and the concentration of these chemical species becomes homogeneous.^7^ The science underlying these phenomena contributes to the understanding of material corrosion in the nuclear industry,^8^ the curative effects of cancer treatment in radiotherapy,^9^ and the mechanisms of DNA damage induction.^10^

**Fig. 1:**
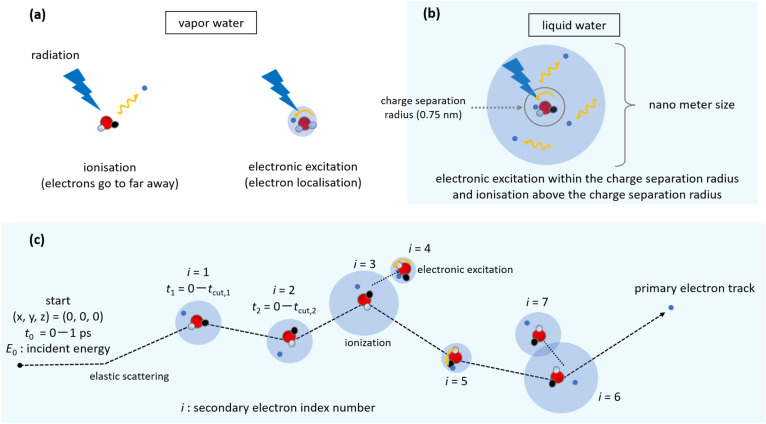
Illustrations of the ionisation and electronic excitation (a) in water vapour and (b) in liquid water. (c) An illustration of the electron track structure.

The pulse-radiolysis technique is employed in experiments on water radiolysis using electron beams. The experiments measure the time evolution of the radiolytic species after picosecond order.^11–14^ The results showed that the initial yield (*i.e.*, *G*-value at 1 ps) of e_aq_^−^ ranged from 4.15 to 4.90 (/100 eV).^11–14^ Meanwhile, Monte Carlo codes (MCC).^15–24^ have been used worldwide to quantify the initial yields and spatial distributions of e_aq_^−^ and other radiolytic species. This simulation method models molecular decay channels, the so-called production branching ratio, of water radiolytic species.^25–30^ In general, the branching ratio (physicochemical model) plays a key role in connecting the physical and chemical processes,^25–30^ although the ratio is determined empirically. As depicted in Fig. 1(a), experimental techniques easily allow a clear distinction between ionisation and electronic excitation in the vapour phase, however, it is still difficult to distinguish ionisation from electronic excitation in liquid water (see Fig. 1(b)).

In this context, we have developed a dynamic Monte Carlo code for physical processes (dmcc_phys) which uses the Monte Carlo method and molecular dynamics method, and thus enable us to derive a robust inference regarding the localisation, relocalisation, delocalisation from the parent molecule, and thermalisation of the secondary electrons resulting from water radiolysis in the order of femtoseconds.^6,31–40^ We have defined both ionised and excited electrons as secondary electrons,^6^ and found that the spatial distribution of the secondary electrons can be classified into a localised component bound to the parent ion (electronic excitation) and a delocalised component ejected from the parent ion (ionisation).^6^ These components are distinguished by the charge separation radius as shown in Fig. 1(b).^6^ From these two components, we predicted an initial *G*-value of e_aq_^−^, *i.e.*, 4.05 (/100 eV), at a primary electron energy of 10 keV.^6^ However, our previous results still contain uncertainties for the charge separation radius and dissociative electron attachment (DEA), which is one of the atomic interaction processes^6^ in which specific low-energy electrons can attach to a molecule and cause its dissociation.

In our previous study,^6^ we introduced the concept of a charge-separation radius to distinguish ionisation from electronic excitation. We also scaled the gas-phase DEA cross section, as no corresponding data for liquid water have been reported. In this study, the DEA cross sections are neglected and the charge separation radius based on a renewed assessment (Fig. 1(b)). We examined the femtosecond dynamics of secondary electrons after irradiation with 20 eV–30 keV electrons in liquid water to investigate the primary electron energy dependence of the initial e_aq_^−^ yields. The results provide an improvement in the long-standing physicochemical models used in water radiolysis and contribute significantly to the seamless connection of physical and chemical processes.

## Results

### Overview

First, we describe the simulation setup, definition and code modification. Second, we show simulation results for penetration range and *G*_total_(*t*_0_), which is the total yield of ionisation and electronic excitation at time *t*_0_ (/100 eV), where *t*_0_ is the flight time from 0 fs to 1000 fs of primary electrons. Third, simulation results for the energy and spatial distributions of the secondary electrons are presented. Finally, *G*_hyd_(1 ps) (/100 eV) and *W* values (eV) for secondary electrons produced by primary electrons with energy of 20 eV–30 keV are presented, where *G*_hyd_(1 ps) is the e_aq_^−^ yield (*i.e.* the number of e_aq_^−^ generated by 100 eV of energy deposition) at 1 ps, and *W* is the average energy required for one ionisation.

### Simulation setup

An electron track is illustrated in Fig. 1(c). The primary electrons of 20 eV–30 keV irradiate from the origin (0, 0, 0) along the *z*-axis direction in this simulation. The dmcc_phys code calculates the dynamic motion of primary and secondary electrons along the main time *t*_0_. Each secondary electron is assigned an index number *i* (=1, 2, …, *N*_e_), and the charges of the *i*-th secondary electron and its parent ion have a dielectric response *ε*_r_(*t*_*i*_) along time *t*_*i*_ from 0 to *t*_cut_,_*i*_, which is cutoff time of each electron of index *i*.^38^*N*_e_ is the number of secondary electrons. Since the MC algorithm was used in this calculation, trial calculations (5 × 10^5^) were repeated until the statistical uncertainty was considerably smaller than 1%.

### Definition

We defined the time-dependent *G*-value of secondary electrons in the femtosecond order. The initial energy of primary electrons is *E*_0_. The total number of ionisation and electronic excitations induced until *t*_0_, *N*_total_(*t*_0_), are obtained using the code. The total-*G*_total_(*t*_0_) value of ionisation and electronic excitation at *t*_0_ is expressed as *N*_total_(*t*_0_)/*E*_0_ × 100 (/100 eV). The ratio of ionisation and electronic excitation determined based on the spatial distribution of secondary electrons at 1 ps are *P*_ion_(1 ps) and *P*_exc_(1 ps), respectively. That is, *P*_ion_(1 ps) + *P*_exc_(1 ps) = 1, since the ionisation yield *P*_ion_(1 ps) is equal to the initial *G* value of e_aq_^−^, which can be obtained by *G*_total_(1 ps) × *P*_ion_(1 ps).

### Code modification

Our code had two assumptions involving uncertainties.^6^ The first one is the charge separation radius, which distinguishes between electronic excitation and ionisation from the spatial distribution of secondary electrons as shown in Fig. 1(b), set to 1.00 nm in our previous study.^6^ However, the reaction radius between H_3_O^+^ and e_aq_^−^ is 0.75 nm.^27^ In this study we changed the radius from 1.00 to 0.75 nm. The other assumption is the DEA yield in liquid water. Since 2000, DEA has attracted attention as one of the new atomic interaction processes that induce DNA damage.^41^ In a previous experiment,^41^ DNA thin films in a vacuum were irradiated with low-energy electrons. We previously used the DEA cross section of water vapour.^6,42,43^ However, it was reported that DEA is less likely to be induced in aqueous solution.^44^ According to a previous simulation using the Geant4-DNA toolkit,^28^ DEA contributes very little to the formation of OH^−^ (initial *G* value 0.02 (/100 eV)). This led us to assume that DEA may be negligible in liquid water. Therefore, DEA is not considered in our simulations. However, there is ample room for debate regarding DEA.

### Penetration range and total *G* value

**Fig. 2:**
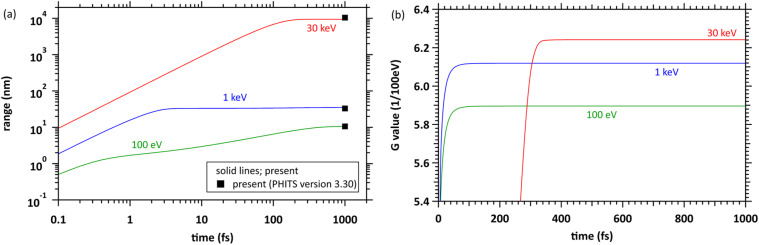
(a) Time evolution of penetration range at primary electron energies of 100 eV, 1 keV, and 30 keV. (b) Time evolution of *G* values 1/(100 eV) of the sum of ionisation and electronic excitation at primary electron energies of 100 eV, 1 keV, and 30 keV.

Fig. 2(a) shows the time evolution of the penetration range for mono-energetic primary electrons with energies of 100 eV, 1 keV, and 30 keV, as a function of time *t*_0_. The penetration range with time evolution is the distance connected by a straight line from coordinate (0, 0, 0) to electron positions at each time point. When the primary electrons have energies of 100 eV and 1 keV, these electrons spread over 10 nm and several 10 nm, respectively, and spread over 10 µm at 30 keV. We also calculated the ranges of the PHITS electron track structure mode (PHITS-ETS)^24^ to confirm the agreement with the conventional time-independent MCC. The PHITS-ETS results are plotted at 1 ps because of the connection to the PHITS original chemistry code (PHITS-Chem) at 1 ps.^25^ These results indicated that dmcc_phys and conventional MCC output similar results for primary electrons. We also show simulation results for *G*_total_(*t*_0_), which counts both ionisation and electronic excitation events, in Fig. 2(b). When the primary electron energies are 100 eV and 1 keV, the inductions of ionisation and electronic excitation are completed within a few 10 fs. When the energy is 30 keV, the inductions continue for about 300 fs.

### Energy and spatial distributions of secondary electrons

**Fig. 3:**
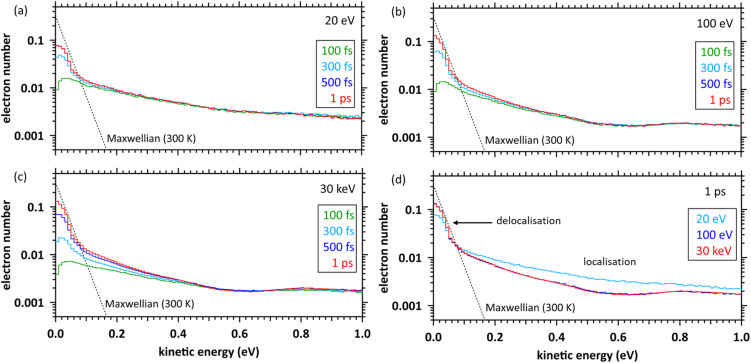
Time evolution of kinetic energy distributions of secondary electrons. (a) Primary electron energy of 20 eV. (b) Primary electron energy of 100 eV. (c) Primary electron energy of 30 keV. (d) Kinetic energy distributions of secondary electrons at 1 ps.

Fig. 3(a)–(c) show the time evolution of the secondary electron energy distributions for primary electrons with energies of 20, 100 eV, and 30 keV, respectively. When the primary electron energies are 20 and 100 eV, the component of the secondary electron energy below 100 meV asymptotically approaches Maxwellian (300 K), which is the energy distribution of non-relativistic classical particles in thermodynamic equilibrium, with time evolution. And the result at 500 fs (blue line hidden by red line) is almost the same as that at 1 ps (red line) since we assumed that thermalised secondary electrons stop and gradually hydrate at the stopped position (see our previous studies,^38,39^ indicating that the secondary electrons are close to be thermalised. When the primary electron energy is 30 keV, the result at 500 fs is not equal to that at 1 ps; the thermalisation time of the secondary electrons seems to get longer. This is due to the results represented by time *t*_0_ until 1 ps, instead of *t*_*i*_ (*i* = 1, …, *N*_e_) until 500 fs. Thus, 30 keV primary electrons continue to induce ionisation and electronic excitation until 300 fs as shown in Fig. 2(b), and each secondary electron (*i* = 1, …, *N*_e_) sufficiently asymptotes to Maxwellian (300 K) within 500 fs. The non-thermal equilibrium component above 100 meV which deviates from the Maxwellian (300 K) in Fig. 3(a)–(c) is a localised component trapped in the coulombic field of the parent ion.^6^Fig. 3(d) shows the energy distributions at 1 ps. The result at 20 eV is higher than the other results above 100 meV. This fact indicates that the incident electrons with energy of a few 10 eV localise generated secondary electrons to parent molecules and would reduce the initial e_aq_^−^ yields.

**Fig. 4:**
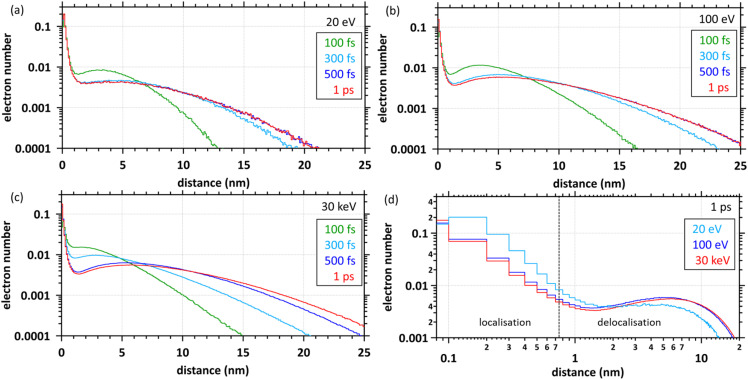
Time evolution of spatial distributions of secondary electrons. (a) Primary electron energy of 20 eV. (b) Primary electron energy of 100 eV. (c) Primary electron energy of 30 keV. (d) Spatial distributions of secondary electrons at 1 ps.

Fig. 4(a)–(c) show the time evolution of the spatial distribution of secondary electrons for primary electrons with energies of 20, 100 eV, and 30 keV, respectively. These results show that the distributions are categorised into two components with time evolution, one for localised (within 0.75 nm) and the other for delocalised (above 0.75 nm). When the energies of primary electrons are 20 and 100 eV, the result at 500 fs is the same as that at 1 ps, indicating that the charge separation of water molecules is complete. When the primary electron energy is 30 keV, the result at 500 fs has not yet converged to the result at 1 ps; the 30 keV primary electrons continue to induce ionisation and electronic excitation until 300 fs as shown in Fig. 2(b), and each secondary electron (*i* = 1, …, *N*_e_) sufficiently asymptotes to Maxwellian (300 K) until 500 fs as shown in Fig. 3(b). Thus, the charge separation is fully complete at 1 ps. Fig. 4(d) shows the spatial distributions at 1 ps. It can be seen that more electrons are localised to the parent molecules with decreasing primary electron energy, reducing the initial e_aq_^−^ yields.

### Initial *G* and *W* values

**Fig. 5:**
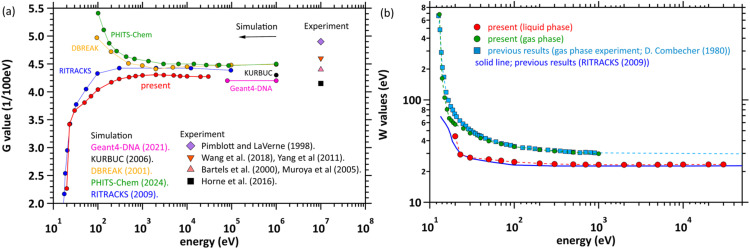
(a) Initial *G*-values for electron irradiation of water. The symbols at 10^7^ eV correspond to experimental values.^7,11–15^ (b) *W*-values for electron irradiation of water.

Fig. 5(a) shows the *G*_hyd_(1 ps) results for primary electron energies of 0.02–30 keV. Note that the experimental data used electron beams of several tens of MeV (categorised as low-LET radiation). The corresponding experimental results are plotted at 10 MeV. The present value of 4.30 (/100 eV) hardly changes above 1 keV. In the comparison with the experiments, the present results are found to be close to those of Bartels *et al.* (4.40 (/100 eV))^11^ and Muroya *et al.* (4.40 (/100 eV)).^12^ The results are slightly higher than those of Horn *et al.* (4.15 (/100 eV)),^7^ but lower than those of Pimblott and LaVerne (4.90 (/100 eV)),^15^ Wang *et al.* (4.60 (/100 eV)),^13^ and Yang *et al.*^14^ (4.60 (/100 eV)). In comparison with previous simulation studies, the present results at about 1 keV are slightly lower than those of the D-BREAK system (4.5 (/100 eV))^45^ and PHITS-Chem (4.5 (/100 eV)),^25^ but similar to those of Geant4-DNA (4.20 (/100 eV))^28^ and KURBUC (4.30 (/100 eV)).^16^ Interestingly, the *G*_hyd_(1 ps) values of dmcc_phys are considerably different from those of the D-BREAK system and the PHITS-Chem code below 1 keV. The increase in the present *G*_hyd_(1 ps) value with increase of the primary electron energy is due to a decrease in secondary electron localisation as shown in Fig. 4(d). Present results are generally close to those of RITRACK.^20^

Fig. 5(b) shows the *W* value (eV), which is the mean energy required for single ionisation and was converted from the *G*_hyd_(1 ps) value (/100 eV) of liquid water. The cross sections for water vapour were also used in the earlier version of dmcc_phys.^31^ The results using the previous dmcc_phys reproduce well those of the water vapour experiments of Combecher.^46^ While the results of the latest version using the liquid-phase cross-sectional data agree well with those calculated by RITRACKS,^20^ they do not reproduce those of water vapour experiments.^45^ Converting the *W* value (29.6 eV) obtained in the experiment in water vapour at 1 MeV to *G*_hyd_(1 ps) yields 3.4 (/100 eV), which does not reproduce that of liquid water. This fact originates from the difference in the energy loss function (or oscillator strength) in the gas and liquid phases.^19^

**Fig. 6:**
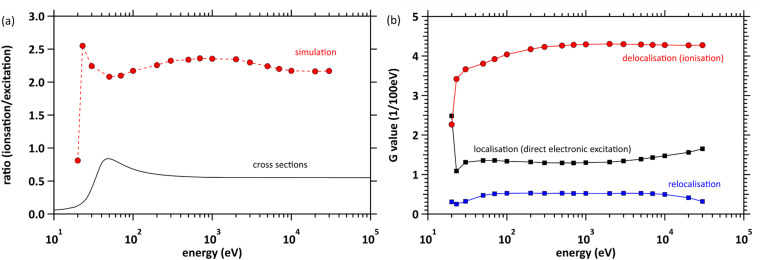
(a) Ratios (total ionisations/total electronic excitations). (b) *G* values (1/100 eV) of delocalisation, localisation and relocalisation.

Fig. 6(a) shows the ratios (total ionisations/total electronic excitations). Present results indicate that ionisation yield is approximately two-fold of electronic excitation yield. However, cross section database shows that the ratio is small because our largest cross section (Fig. S1(a) in the SI) is assigned to collective excitation referred to literature of Paretzke *et al.*^18,47^ Thus, the ratio also depends strongly on the assignment of various energy levels. Generally, the deposition energy exceeding the ionisation energy (10.9 eV (ref. 48)) to water results in H_3_O^+^, ˙OH and e_aq_^−^*via* H_2_O^+^ and e^−^. These initial yields are equal. However, water photolysis experiments^49^ reported that the initial ratio [˙OH]/[e_aq_^−^] is 1.1 at the deposition energies of 12.4 eV. This experimental evidence suggested that relocalisation (and dissociation following relocalisation) is more likely the cause of the discrepancy here. We interpreted this pathway as relocalisation. Fig. 6(b) shows the *G* values (1/100 eV) of delocalised, localised and relocalised electrons. These results indicate that relocalisation is approximately one-third and one-eighth of localisation (direct electronic excitations) and delocalisation, respectively. From the results at 20 eV, we found that the increase of localisation as shown in Fig. 4(d) originated from direct electronic excitations.

## Discussion

### Code validation

Although the initial *G*-value of e_aq_^−^ in the low-energy region has not been reported, the thermalization distances 1.0–8.5 nm in injection electron energy from 0.1 to 4.0 eV (ref. 50) and the spur radii 0.8–4.0 nm of secondary electrons in total photon energy from 8.3 to 12.4 eV^51–56^ have been reported. Our previous studies reproduce these experimental values, sufficiently validating this code.^6,33,37,40^ The validation indicated that delocalised components of the secondary electrons at the nanometre scale can be demonstrated by the code, but challenges remain regarding localised components at the angstrom scale, such as bound electrons. The total yield of ionisation and electron excitation in water depends on the cross section derived from the energy loss function (ELF) measured experimentally.^57^ Our previous^6,33,37,40^ and present studies demonstrated that ionisation process with electron delocalisation at nanometre scale can be simulated by the code even in the low-energy region.

### Necessity of electron dynamic motion

Accurate assessment of the initial *G* values of radiolytic species is crucial for estimating the indirect effects of DNA damage, as the type of DNA damage produced by indirect effects depends on the kind of chemical species.^58^ Many conventional MCCs use electron impact cross sections of water vapour. This may be due to the simple formulation of the ionisation and electronic excitation cross sections.^16^ As shown in Fig. 5(b), it is possible to reproduce the *W* value^46^ in water-vapour experiments by using these cross sections. However, it is not possible to reproduce *G*_hyd_(1 ps) in liquid water without the physicochemical models.^25–30^ In those models, some secondary electrons are ejected when specific electronic excitations are induced.^25–30^ These models parameterise the ratio for the dissociation of excited molecules so that the time-evolution yields of the radiolytic chemical species match the experimental values in the chemical process. These models basically have been employed for several decades, although the branching ratios have updated over the years.^59^

In this study, based on localisation, relocalisation, and delocalisation simulations of secondary electrons, the *G*_hyd_(1 ps) value was estimated at primary electron energies of 20 eV–30 keV. Above 1 keV, we achieved the theoretical prediction of *G*_hyd_(1 ps) 4.3 (/100 eV), a well-known value. The *G*_hyd_(1 ps) value of dmcc_phys significantly differed from that of MCC below 1 keV, gradually highlighting electron localisation with decreasing primary electron energy, as shown in Fig. 4(d). Therefore, some conventional physicochemical models need correction for the energy dependence of primary electrons. The present study proposes that it is not essential to precisely estimate the cross-sectional ratios of ionisation and electronic excitation assigned by various discrete levels in liquid water when limited to initial *G* values of e_aq_^−^; instead, the dynamic motion calculation of secondary electrons determines the final ratio of total ionisation to total electronic excitation. In this way, we must depart from traditional physicochemical models. Future work should address the significant challenges associated with molecular dissociation *via* electronic excitations, as the current version of our code does not yet reproduce bound orbitals within a quantum-theoretical framework.

### Charge separation radius

When the spur size of e_aq_^−^ is extremely small, the escape yield of e_aq_^−^ becomes highly sensitive to this radius.^49^ Consequently, the charge separation radius within 1 nm is a critically important parameter that strongly influences subsequent chemical processes and therefore requires careful consideration. In our previous study, this radius was set to 1 nm based on the shape of the electron spatial distribution.^6^ However, we later found that the reaction radius between e_aq_^−^ and H_3_O^+^ is 0.75 nm,^27^ implying that electrons located more than 0.75 nm away from the cation can potentially undergo hydration. We therefore reconsidered the charge separation radius and set it equal to the reaction radius. This approach appears reasonable, as our results agree well with the previous results^7,11–16,20,22,25,45^ in the high-energy region. Changing the charge separation radius to 0.75 nm led to an increase of 0.2 (1/100 eV) in the *G* values a change that is not negligible and could affect subsequent chemical reactions. This finding is expected to be useful when extending this method to other solvents and may ultimately serve as a new general concept in the physicochemical stage, bridging radiation physics and chemistry.

### Dissociative electron attachment

The DEA has been measured by irradiating a water molecule in vacuum.^42^ It is well known that DEA is a resonance phenomenon with the vibrational excitation of molecules. When electrons approach water molecules in a vacuum, anion formation is possible at a specific incident energy. Here, low-energy electrons attached to water molecules can induce vibrational excitations by collisions. When this vibrational excitation is induced repeatedly, and the vibrational energy in a single molecule is amplified, finally, the target molecule dissociates when the amplified vibrational energy exceeds its dissociation energy. When attached electrons are desorbed before the molecule dissociates, the resulting vibrational excitation cross section with a resonance structure is measured.^60^ Here, we consider the phenomenon induced in the liquid phase. It would be possible for electrons to attach to water molecules in the liquid phase. This is because DEA also seems to be induced in amorphous ice-thin films in a vacuum.^61^ However, in the liquid phase, it is possible that other surrounding water molecules overlap within the wave functions of the attached electrons. The kinetic energy of the attached electrons would not only amplify the vibrational energy of a specific attached water molecule but also amplify that of the surrounding multiple water molecules. Thus, the energy required for dissociation would be dispersed, and the DEA yield might be severely reduced. In fact, a decrease in DEA yield has been experimentally observed when water molecules are gradually coordinated with biomolecules.^61^ Recent simulation results indicated that molecular dissociation of thymine *via* DEA is affected by interactions with the surrounding water molecules when the base is in an aqueous environment.^62^ It was also reported that DEA is less likely to be induced in liquid water.^44^ Thus, we hypothesised that the kinetic energy of the attached electrons is converted to vibrational energies of the surrounding multiple molecules. In this case, the amplified vibrational energy does not exceed the dissociation energy, resulting in an extreme decrease in DEA yield. It would also be interesting to investigate the primary electron energy dependence of initial *G*-values of e_aq_^−^ to show the difference between our simulation code (dmcc_phys) and the conventional MCCs.

### Initial *G* values

As shown in Fig. 5(a) for liquid water, our results exhibit an incident electron energy dependence that differs from some previous ones.^25,45^ No experimental values exist for the initial *G* values of e_aq_^−^ for the low-energy region of liquid water. As shown in Fig. 5(b) for gas-phase water, experimental values for the *W* value have been reported in the energy region above 12.7 eV.^46^ The *W* value increases as the incident electron energy decreases. Our gas phase results agree well with the experimental values. Converting those *W* values to *G* values shows that the *G* values decrease as the incident electron energy decreases. A similar trend was obtained for liquid phase as shown in Fig. 5(a), making our results reasonable. RITRACKS^20^ also shows a similar trend. The e_aq_^−^ yields remain constant above 1 keV, while decreases below 1 keV. As the incident electron energy decreases, the *G* values decrease since possible deposition energy decreases.^39^ Consequently, transition of the lower-electronic excitation (localisation component) increases at 20 eV as shown in Fig. 6(b), whilst the ionisation (e_aq_^−^) considerably decreases.

### Hydration

According to our previous study,^37^ electronic polarisation responds instantaneously on an attosecond scale, phonon polarisation responds from a few 10 fs to a few 100 fs, and orientation polarisation strongly dominated the dielectric response after a few 100 fs. The dielectric response showed a maximum value of 80 after a few tens of picoseconds. No results have been reported for the spatial distribution of decelerating secondary electrons calculated by quantum theory. Although we solved classical electron motion, the electron propagation strongly depends on cross sections based on quantum theory. Our previous study demonstrated that electron thermalisation is approximately complete within several hundred fs.^37–39^ Subsequently, the orientation polarisation increases sharply after a few 100 fs. Hydration simulation requires quantum effects. The simulation was performed using a mixed quantum mechanics/molecular mechanics (QM/MM) dynamical approach.^63^ The results indicated that the diffusion motion of electrons is strongly restricted during the hydration.^63^ Thus, the distribution of thermalised electrons at several hundred fs is nearly equal to the initial distribution of e_aq_^−^. The conventional method uses the same approach.^25^ As electron hydration progresses, the coulombic fields of parent ions also decrease. Using our code, we calculated time evolution of potential energy produced by the parent ion at 0.75 nm. The energy is 0.27, 0.22 and 0.15 eV at 300, 500 and 1000 fs, respectively. In contrast, according to experimental data,^64–66^ the vertical detachment energy, which is the energy required to remove an electron from the chemical species, of e_aq_^−^ is 3.3–3.6 eV. Consequently, a part of the electrons localised by the coulombic field at several 100 fs are gradually hydrated by surrounding water molecules. Thus, the charge separation radius gradually decreases.^67^ Ultimately, the radius will approach the classical Onsager radius (0.72 nm),^68^ which is nearly equal to the reaction radius between H_3_O^+^ and e_aq_^−^ (0.75 nm). Therefore, we adopted the experimentally determined reaction radius as the final charge separation radius that distinguishes ionisation from electronic excitation.

When the incident electron energy decreases below 10 eV, molecular excitations become dominant over electron excitations (Fig. S1 in SI),^6,39^ and the epithermal electrons gradually close to thermal electrons *via* many molecular excitations and elastic scattering.^37^ To simulate the subsequent hydration process, a dielectric response function was derived.^37^ This dielectric response governs the relocalisation yields of secondary electrons. The relocalisation rates are very high when the deposition energy to water is 11 eV,^38^ however they decrease dramatically once when the deposition energy exceeds 13 eV.^38^ Our code implements this dielectric response function (Fig. S2(a) in SI), inducing phonon polarization within tens of fs, orientation polarization within hundreds of fs, and completing the polarization effect within tens of ps.^37^

### Scientific advances and future

Our dmcc_phys enables the prediction of *G*_hyd_(1 ps) by a computer simulation without physicochemical models, and provides a new approach to water radiolysis based on new physical findings for *G*_hyd_(1 ps). We expect that it will gradually become recognised as a good simulation code for water radiolysis. The present code can calculate electron localisation, relocalisation, delocalisation, and thermalisation resulting from water radiolysis in the order of femtoseconds. After this charge separation within a few 100 fs is sufficiently complete, hydration is in full swing,^37^ and the hydration is complete after several tens of ps.^37^ Therefore, we propose that when liquid water is irradiated with electron beams, the water radiolysis changes into the charge separation phase (ionisation, electronic excitation, and thermalisation), hydration phase in the femtosecond order,^1–5,37^ full-scale hydration phase from 1 ps to several tens of ps,^37^ and chemical reaction phase after several hundred ps.^25–30^ In this study, the *G*_hyd_(1 ps) values were calculated at primary electron energies of 20 eV–30 keV. Experimental values for the time-dependent *G*-values of water radiolytic products can be traced *via* simulation.^25–30,45^ Those values depend strongly on the initial *G* values and initial positions (1 ps) of the products. Therefore, we have continued the intensive development of physical^6,31–40^ and chemical code.^67,69^ Connecting these in the future will enable time-dependent *G*-value simulation of e_aq_^−^. Comparing the simulation with experimental results would allow us to demonstrate our finding in the future work. In the pulse-radiolysis technique, water is irradiated with electrons with energy of several tens of MeV. This requires us to present simulation results for 1 MeV electrons. We will calculate *G*_hyd_(1 ps) until 1 MeV, and also explore the formation mechanisms of other chemical species, such as ˙OH radicals. We also need to connect dmcc_phys with our chemistry code, dmcc_chem,^67,69^ to validate the new concept, which will lead to a precise understanding of the unknown physicochemical process. Finally, further developments will contribute to deeper understanding in the nuclear industry,^8^ radiotherapy,^9^ and radiation biology,^10^ such as the mechanisms of DNA damage induction by indirect effects.^69^

## Conclusions

This study assumed that the charge separation radius is 0.75 nm, and that DEA is negligible in liquid water. We investigated the femtosecond dynamics of secondary electrons after irradiation with electrons ranging from 20 eV to 30 keV in liquid water to study the primary electron energy dependence of the initial *G*_hyd_(1 ps) value without using conventional physicochemical models. We compared the *G*_hyd_(1 ps) values to those of experiments and simulation studies in the literature. Although we reproduced well-known values above 1 keV, the *G*_hyd_(1 ps) values of dmcc_phys were considerably different from those of some MCC below 1 keV due to an increase of the secondary electron localisation with decreasing primary electron energy. Radiation can excite or ionise water molecules into various electronic states. These transitions can be explained by the ELF (or oscillator strength). Unlike isolated water molecules, the ELF of liquid water does not exhibit a discrete spectrum but rather a broad continuous spectrum with a maximum near 21 eV.^19^ Consequently, bound electrons in molecules readily transition to highly excited states. A part of highly excited electrons may collide with water molecules surrounding the parent cations^38^ and hydrate after electron thermalisation.^37–39^ In fact, previous studies have indicated that electrons attaching to biomolecular anions in water are affected by surrounding water molecules.^62,63^ This case is ultimately judged as ionisation in the physicochemical stage after a few hundred femtoseconds (fs), even if highly excited electrons are produced in the physical stage within a few femtoseconds. This clarification and evaluation are the objective of this study. Before achieving this objective, our previous studies validated the spatial distribution calculation method for extremely low-energy electrons by comparing it with experimental values over many years.^6,33,37,40^ Our method also reproduces the initial *G* value of e_aq_^−^ of previous results.^7,11–16,20,22,25,45^ When limited to simulating the e_aq_^−^ generation, we cannot precisely define the cross sections of ionisation and electronic excitation assigned by various levels in liquid water. We propose that the dynamic motion calculation of secondary electrons determines the final ratio of total ionisation to total electronic excitation. Our method is also expected to provide initial yields and spatial distributions for radiolytic chemical species other than e_aq_^−^, contributing to various research fields involving water radiolysis.

## Author contributions

T. Kai and T. Toigawa designed this work. T. Kai developed the dynamic Monte Carlo code for the physical process and performed all calculations, except for the PHITS calculation. Y. Matsuya performed the PHITS calculation. Y. Matsuya and Y. Hirata contributed to the discussion for the development of the code and radiation physics. H. Tsuchida contributed to the discussion of radiation physics. T. Toigawa contributed to the discussion of radiation chemistry. A. Yokoya supervised this study. T. Kai wrote the manuscript. All authors contributed to the discussion of this study and have reviewed the manuscript.

## Conflicts of interest

There are no conflicts to declare.

## Supplementary Material

RA-016-D6RA00147E-s001

## Data Availability

The data supporting the findings of this study are available from the corresponding author upon reasonable request. Supplementary information (SI): all simulation methods for dmcc_phys (cross section, dielectric response, time-dependent MC method, MD method and calculation flowchart) were written in a SI file. See DOI: https://doi.org/10.1039/d6ra00147e.
