# Supplementary material for: An initial G value of hydrated electrons updated by a dynamic Monte Carlo simulation
Source: RSC Adv. 2026 Mar 13;16(15):13886–95. doi: 10.1039/d6ra00147e (PMC12983369; doi:10.1039/d6ra00147e)
Supplement: RA-016-D6RA00147E-s001 [file RA-016-D6RA00147E-s001.pdf]

Supplementary Information (SI) for RSC Advances.
This journal is © The Royal Society of Chemistry 2026

Supplementary information

An initial G value of hydrated electrons updated by a dynamic Monte Carlo simulation

Takeshi Kai[1], Tomohiro Toigawa[1], Yusuke Matsuya[1,2], Yuho Hirata[1], Hidetsugu Tsuchida[3,4], Akinari Yokoya[5]

[1]*Nuclear Science and Engineering Center, Japan Atomic Energy Agency, 2-4 Shirane Shirakata, Tokai-mura, Naka-gun, Ibaraki, 319-1195, Japan*

[2]*Faculty of Health Sciences, Hokkaido University, Kita-12 Nishi-5, Kita-ku, Sapporo, Hokkaido 060-0812, Japan*

[3]*Department of Nuclear Engineering, Kyoto University, Nishikyo-ku, Kyoto 615-8530, Japan*

[4]*Quantum Science and Engineering Center, Kyoto University, Gokasho, Uji, Kyoto 611-0011, Japan*

[5]*Institute for Quantum Life Science, National Institutes for Quantum Science and Technology, 4-9-1 Anagawa, Inage-ku, Chiba-shi 263-8555, Japan*

Methods

We published the first version of dmcc_phys in 2014 [1], which used cross-sectional data for water vapour [1]. Calculation of molecular excitation cross sections for liquid water at 1 meV–100 eV [2] enabled us to analyse electron dynamics in the extremely low energy region on the order of meV [3]. As an example of our results, we have confirmed that the energy distribution of secondary electrons resulting from water radiolysis and photolysis asymptotically approaches Maxwellian (300 K) due to molecular excitation and momentum transfer by elastic scattering [4–6]. In this study, all low-energy electrons generated are referred to as secondary electrons. The spatial distribution of secondary electrons is composed of a localised component (electronic excitation) bound to the parent ion and an ejected delocalised component (ionisation) [4]. From this delocalised component, the initial yield of hydrated electrons is predicted by a computer simulation [4]. The dielectric response of water was also obtained by Fourier transformation of the complex dielectric function of water [4,5].

The dmcc_phys outputs the spatial and energy distributions of secondary electrons at each time using the time-dependent MC and molecular dynamics (MD) methods [4–6]. Therefore, in our code, the cutoff time needs to be set instead of the cutoff energy generally needed to be set in general MCCs such as Geant4-DNA and PHITS track-structure mode. This allows the code to calculate the dynamic motions of secondary electrons, which collide with water molecules and move in the Coulombic field created by the parent ion from moment to moment. As a result, it

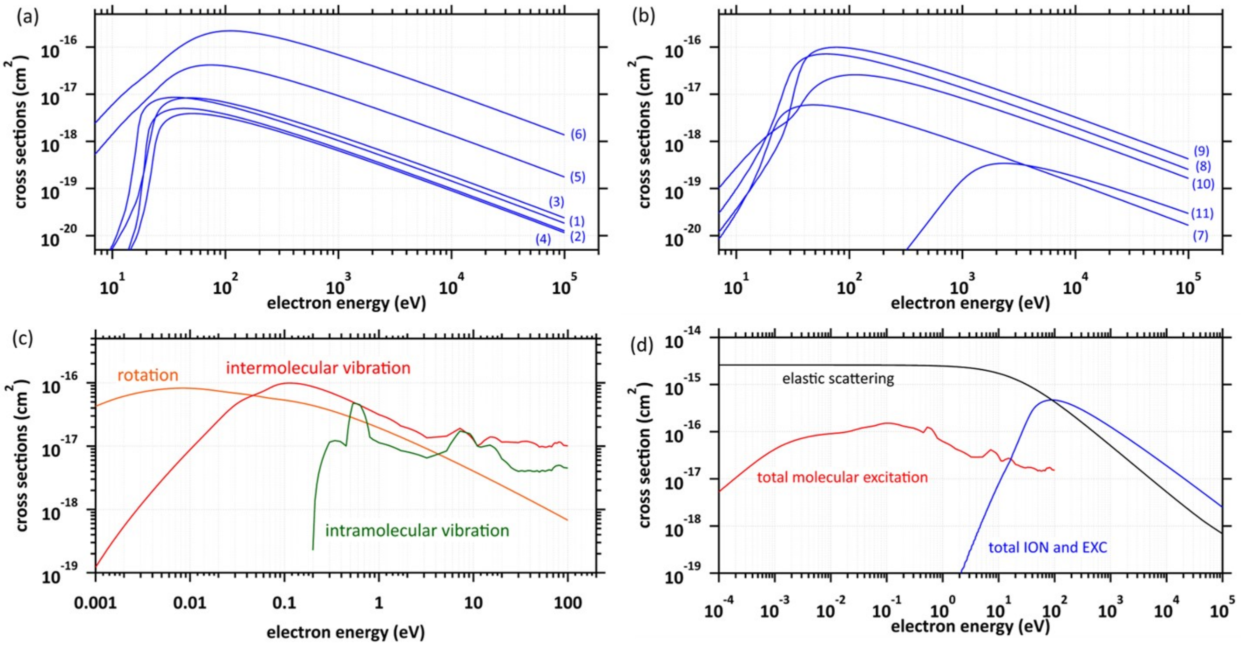

Fig. S1 (a) Cross-sections in the energy range from 10 eV to 100 keV; (1) A^1B_1 excitation (8.4 eV); (2) B^1A_1 excitation (10.1 eV); (3) Rydberg (A + B) excitation (11.25 eV); (4) Rydberg (C + D) excitation (11.93 eV); (5) diffuse band excitation (14.1 eV); (6) collective excitation (21.4 eV). The energy in parentheses represents the mean value of the transition energy assigned by Paretzke [7]. (b) Cross-sections in the energy range from 10 eV to 100 keV; (7) $1b_1$ ionisation (10.9 eV); (8) $3a_1$ ionisation (13.5 eV); (9) $1b_2$ ionisation (17.0 eV); (10) $2a_1$ ionisation (26.3 eV) in the energy range from 10 eV to 100 keV; (11) $1a_1$ ionisation (533 eV). The energy in parentheses represents the mean value of the transition energy assigned by Faubel [8]. (c) Rotation, inter- and intra-molecular vibration excitation cross-section [2]. (d) Elastic scattering cross section [9], total molecular excitation cross sections, and total ionisation (ION) and electronic excitation (EXC) cross sections.

is possible to calculate the localisation, relocalisation, delocalisation, and thermalisation of the electrons. Furthermore, the hydration process is simulated by shielding this Coulombic field along the dielectric response [5]. In this section, the cross section, dielectric response, time-dependent MC and MD methods, and the flowchart in dmcc_phys are briefly described.

Cross section

The electronic excitation and ionisation cross sections of the condensed phase are calculated using the energy loss function, which is obtained from the complex dielectric function [10]. Measurements of the complex dielectric function of water have already been reported [11,12], as well as its fitting parameters [12,13]. We used those fitting parameters to calculate each cross section. Figures S1(a) and (b) show the calculated electronic excitation and ionisation cross sections up to 7 eV–100 keV, respectively. Figure S1(c) shows the results for molecular excitation cross sections. Figure S1(d) shows the results for the total molecular excitation cross section, the total electronic excitation and ionisation cross section, and the elastic scattering cross section.

The differential and integral cross sections $q(\theta)$ and σ_{Elas} of elastic scattering were obtained using Moliere's equations

in Eqs. (1) and (2), respectively [9].

$$q(\theta) = \pi Z(Z+1) r_e^2 \frac{1-\beta^2}{\beta^4} \frac{1}{(1-\cos\theta+2\eta)^2}, \quad (1)$$

and

$$\sigma_{elas} = \pi Z(Z+1) r_e^2 \frac{1-\beta^2}{\beta^4} \frac{1}{\eta(\eta+1)}, \quad (2)$$

where, the shielding parameter η is given by:

$$\eta = \eta_c \times 1.7 \times 10^{-5} Z^{2/3} \frac{1}{\tau(\tau+1)},$$

where, θ is the scattering angle, Z is the effective atomic number of a water molecule, assumed to be 7.42, r_e is the classical electron radius, 2.8179 × 10^{-13} cm, and β is the ratio of the speed of the electron to the speed of light. τ is E/m_0c^2, where E, m_0, and c are the electron energy, rest mass, and speed of light. η_c is given by:

$$\eta_c = 1.198 \quad \text{for } E < 50 \text{ keV},$$

$$= 1.13 + 3.76\left(\frac{Z}{137\beta}\right)^2 \quad E > 50 \text{ keV}.$$

When elastic scattering is induced, no energy change occurs concerning relative motion, but the energy changes concerning centre-of-mass motion [14,15]. This phenomenon, which becomes effective in the very low energy region, is evaluated by the momentum transfer cross section σ_{mom}, which is determined from the differential cross section $q(\theta)$ of elastic scattering [14,15]:

$$\sigma_{mon} = 2\int_0^{\pi} (1-\cos\vartheta) q(\vartheta) \sin\vartheta d\vartheta, \quad (3)$$

where, the energy transfer ΔE is expressed as follows [4–6,14,15]:

$$\Delta E \cong \frac{2m\sigma_{mom}}{M\,\sigma_{Elas}} (E_e - E_{mol}), \quad (4)$$

where, m and M, and E_e and E_{mol}, are the masses and energies of electrons and water molecules, respectively. Here, E_{mol} is sampled from the Maxwellian at 300 K. Eq. (4) means that when ΔE is positive, the electrons supply energy to water, and when ΔE is negative, the electrons are supplied energy by water. Although ΔE is only a few μeV, the secondary electrons asymptotically approach Maxwellian (300 K) after a huge number of these collisions [4–6].

Dielectric response

The complex dielectric function $\varepsilon(\omega)$ of a solvent derives the dielectric relaxation in macroscopic space by the Fourier transform. $\varepsilon(\omega)$ of water has been reported as a function of frequency (Hz) by X-ray, far-infrared, and microwave spectroscopy [11,12]. The complex dielectric function $\varepsilon(\omega)$ of water involved in thermalisation, delocalisation, and hydration of secondary electrons, which is the focus of this study, is given by [12]:

$$\varepsilon(\omega) = \frac{\Delta\varepsilon_1}{1+i\omega\tau_1} + \frac{\Delta\varepsilon_2}{1+i\omega\tau_2} + \frac{A_T}{\omega_T^2 - \omega^2 + i\omega\gamma_T} + \frac{A_L}{\omega_L^2 - \omega^2 + i\omega\gamma_L} + \varepsilon_\infty. \quad (5)$$

We assumed that the dielectric response in microscopic space around electrons and cations in water is also given by

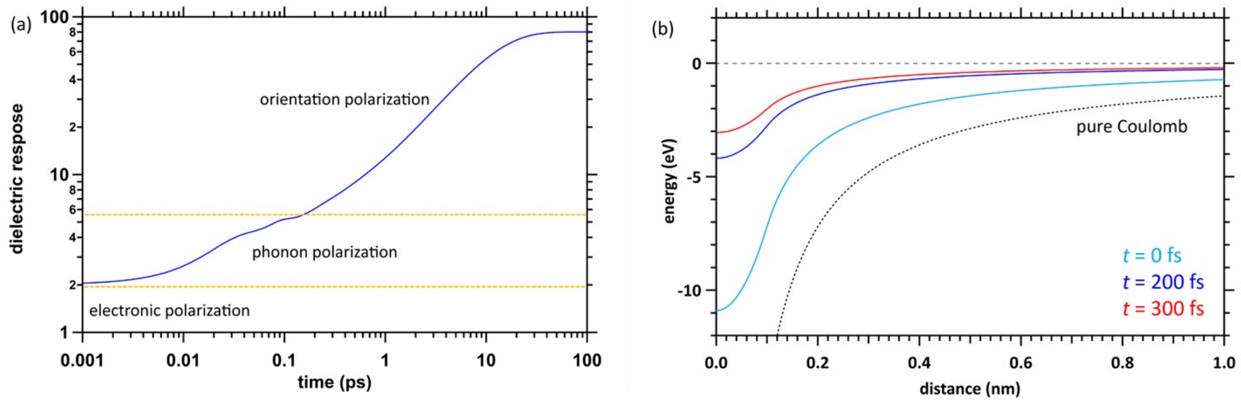

Fig. S2 (a) Time evolution of the dielectric response of water. (b) Time evolution of potential energy assumed in this study.

Fourier transformation. The rotational mode described by the Debye function corresponds to exponential relaxation (first and second terms of Eq. (5)), and the intermolecular vibrational mode described by the Lorentz function corresponds to vibrationally damped relaxation (third and fourth terms of Eq. (5)). From the correlation between relaxation and response, we assumed that the dielectric response $\varepsilon_r(t)$ after charge generation in water can be expressed as follows [5]:

$$\varepsilon_r(t) = \Delta\varepsilon_1\left(1 - \exp\left(-\frac{t}{\tau_1}\right)\right) + \Delta\varepsilon_2\left(1 - \exp\left(-\frac{t}{\tau_2}\right)\right) + \frac{A_T}{{\omega_T}^2}\left(1 - \cos\left(\omega_T t\right)\exp\left(-\frac{\gamma_T t}{2}\right)\right) + \frac{A_L}{{\omega_L}^2}\left(1 - \cos\left(\omega_L t\right)\exp\left(-\frac{\gamma_L t}{2}\right)\right) + \varepsilon_\infty, \quad (6)$$

where $\Delta\varepsilon_1$ and $\Delta\varepsilon_2$ are the intensities of Debye relaxation modes with relaxation times of τ_1 and τ_2 (orientation polarisation) [12]. A_{T} and A_{L} are amplitudes, ω_{T} and ω_{L} are the angular frequencies, and γ_{T} and γ_{L} are the damped vibrations of the intermolecular vibration modes (phonon polarisation) [12]. ε_∞ is the dielectric constant at the high-frequency limit (electronic polarisation) [12]. Here, $\tau_1 = 1.08((T/228) - 1)^{-1.73}$ ps, and the fitting parameters for water at 293 K are $\Delta\varepsilon_1 = 74.9$, $\Delta\varepsilon_2 = 1.67$, $\varepsilon_\infty = 2.0$, $\tau_2 = 248$ fs, $A_T/(2\pi)^2 = 31.5$ THz^2, $\omega_T/2\pi = 5.30$ THz, $\gamma_T/2\pi = 5.35$ THz, $A_L/(2\pi)^2 = 108$ THz^2, $\omega_L/2\pi = 14.7$ THz, and $\gamma_L/2\pi = 8.08$ THz [12]. These parameters are expressed in terms of angular frequency using 2π. Figure S2(a) shows the calculated dielectric response. The results show that after charge generation in water, phonon polarisation is dominant from ~10 fs to ~100 fs, and after ~100 fs, orientation polarisation becomes dominant [5]. Then, after several tens of ps, the dielectric response is complete [5].

Time-dependent MC method

The typical time-independent MCC gives the distance of one step that an electron travels as $\Delta s = -\lambda\ln(k)$ [13], where λ is the mean free path, obtained from the total cross-section σ and the molecular density N (3.318565×10^{22} molecules/cm^3) as $\lambda = 1/(\sigma N)$. k is a uniform random number. This code assumes that electron-water collisions are induced when the following conditions of Eq. (7) are satisfied [4–6]:

$$1 - \exp\left(-\frac{\Delta s}{\lambda}\right) > k, \quad (7)$$

where, $\Delta s = v\Delta t$, v is the absolute value of the velocity, and Δt is the time mesh, set to 1 attosecond. After the collision

position is determined, the collision process is identified. The process is sampled from the cross-sectional ratios of ionisation and electronic excitation, molecular excitation, and elastic scattering, as shown in Fig. S1. If elastic scattering is induced, the scattering angle is sampled from the differential cross-section $q(\theta)$ [9]. From Fig. S1(d), since molecular excitations, which are much less frequent than elastic scattering, are induced [4], our code assumes that the scattering angle does not change when molecular excitations are induced. When ionisation or electronic excitation is induced, the scattering angles θ_1 and θ_2 for primary and secondary electrons are given by the energy and momentum conservation laws, respectively, as in Eq. (8) [13,16]:

$$sin^2\theta_1 = \frac{E_2/E_0}{\left(1 - E_2/E_0\right)E_0/\left(2m_0c^2\right) + 1},\ sin^2\theta_2 = \frac{1 - E_2/E_0}{1 + E_2/\left(2m_0c^2\right)}, \tag{8}$$

where, E_0 and E_2 are the kinetic energies of primary and secondary electrons, m_0 is the rest mass of an electron, and c is the speed of light. The azimuthal angle φ is sampled from a uniform random number. This determines the post-collision velocity vector. Secondary electrons are emitted from various molecular levels. We determined the initial energy E_2 of secondary electrons as shown in Eq. (9).

$$E_2 = W - \left(I_p - \Phi(0)\right). \tag{9}$$

where, deposition energy W is sampled from the energy loss function [6] corresponding to the selected ionisation or excitation with its transition energy I_p. $\Phi(0)$ is first ionisation energy of 10.9 eV.

MD method

This code assumes that electrons and cations are finite-size particles of radius a with negative and positive charge e (finite-size particle model) [4–6]. The potential of the cation is expressed in spherical coordinates as follows:

$$\Phi(r) = \frac{1}{4\pi\varepsilon}\int_{-\infty}^{\infty}\frac{e}{|r - r'|}dr' = \frac{e}{4\pi\varepsilon r}\,(r \geq a)\ \ or\ \ \frac{e\left(3a^2 - r^2\right)}{8\pi\varepsilon a^3}\,(r < a), \tag{10}$$

where, e is the elementary charge. The particle radius a is set to be 0.099 nm to reproduce the ionisation energy of 10.9 eV [8]. ε is expressed as $\varepsilon_0 \times \varepsilon_r(t)$, where ε_0 is the dielectric constant of a vacuum and $\varepsilon_r(t)$ is the dielectric response in Eq. (6). This contributes to simulating hydration, as shown in Fig. S2(b) [4–6]. The produced secondary electrons with initial kinetic energy E_2 are coordinated to $r = 0$ in Eq. 10. The primary electron transport calculation and the change in the velocity vector of the electron due to the dynamic Coulombic field are obtained by solving the relativistic Newton equation in Eq. (11) [4] as follows:

$$\frac{m}{\gamma 3/2}\frac{d}{dt}v_i = \sum_{j \neq i} F_{ij},\quad \frac{d}{dt}x_i = v_i, \tag{11}$$

where,

$$F_{ij} = \pm\frac{e^2}{4\pi\varepsilon r_{ij}^3}r_{ij}\ \ (|r| \geq a), F_{ij} = \pm\frac{e^2}{4\pi\varepsilon a^3}r_{ij}\ \ (|r| < a)$$

Also, $\mathbf{x}_i$ and $\mathbf{v}_i$ are the position and velocity vectors of the i-th electron, and γ is $(1 - v_i^2/c^2)$. This means that the velocity vector of the secondary electron is governed by the collision (Eqs. (1) and (8)) and the dynamic Coulombic

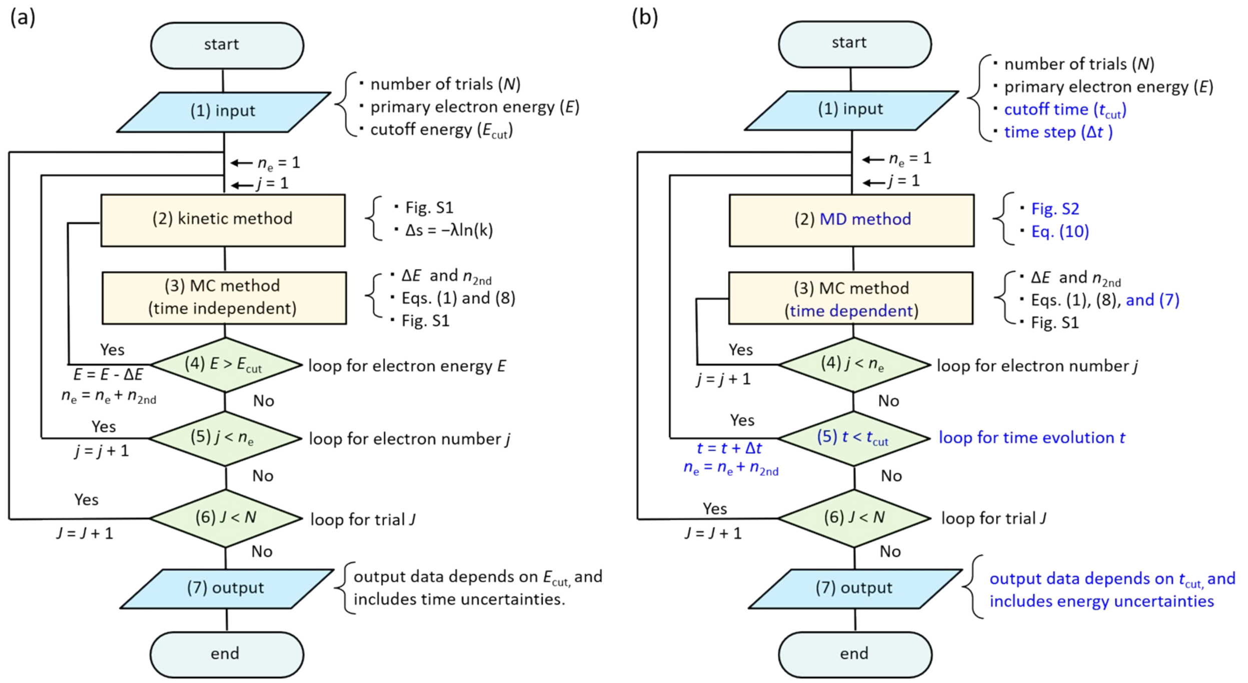

Fig. S3 (a) Flowchart of typical Monte Carlo codes (MCCs) for simulating water radiolysis. (b) Flowchart of dmcc_phys for simulating water radiolysis.

field (Eqs. (9) and (10)). We also assume that within the simulation limit of 1 ps, no radiolytic chemical species other than secondary electrons diffuse thermally on the order of femtoseconds.

Calculation flowchart of dmcc_phys

To clarify the difference between the typical MCC and dmcc_phys, Fig. S3(a) and (b) show flowcharts for both [4–6]. First, we explain the MCCs such as PHITS and Geant4-DNA. (1) The number of trials (N), the initial energy of primary electrons (E_0), and the cutoff energy of electrons (E_{cut}) are input to the MCC. The initial energy of the secondary electron is determined by $E_2 - I_e$ (ionisation energy 10.9 eV [8]). (2) In the kinetic method, electrons are transported by $\Delta s = -\lambda \ln(k)$. (3) In the MC method, the electron losses ΔE and the number of secondary electrons generated n_{2nd} are obtained. Processes (4)–(5) are repeated until the primary and secondary electron energies reach the cutoff energy. (6) After these calculations are completed, we then move on to trial $J = J + 1$. (7) When the statistical error of the calculation results is sufficiently small, all calculations are complete. The calculation results of the general MC code depend on the cutoff energy, and so contain time uncertainties.

Our code takes as inputs (1) the number of trials (N), the initial energy of the primary electron (E_0), the cutoff time (t_{cut}), and the time step Δt (1 attosecond). (2) The dynamic motions of primary and secondary electrons are solved simultaneously for each time step Δt according to the MD method expressed in Eq. (10). (3) In the time-dependent MC method, the collision of electrons with water is determined using Eq. (7), and if collision occurs, the electron losses ΔE and the number of secondary electrons n_{2nd} generated are obtained. After process (3) is completed, we move to the next time $t = t + \Delta t$. Processes (4)–(5) are repeated until the cutoff time is reached. (6) After these

calculations are completed, we move to the next trial $J = J + 1$. (7) All calculations are completed when the statistical uncertainty of the calculation results is sufficiently small. The calculation results depend on the cutoff time, and so contain energy uncertainties.

References

1. T. Kai, A. Yokoya, M. Ukai, K. Fujii, M. Higuchi and R. Watanabe, *Radiat. Phys. Chem*. 2014, **102**, 16-22.
2. T. Kai, A. Yokoya, M. Ukai and R. Watanabe, *Radiat. Phys. Chem*. 2015, **108**, 13-17 (2015).
3. T. Kai, A. Yokoya, M. Ukai, K. Fujii and R. Watanabe, *Radiat. Phys. Chem*. 2015, **115**, 1-5.
4. T. Kai, T. Toigawa, Y. Matsuya, Y. Hirata, T. Tezuka, H. Tsuchida and A. Yokoya, *RSC adv*. 2023, **13**, 7076–7086.
5. T. Kai, T. Toigawa, M. Ukai, K. Fujii, R. Watanabe and A. Yokoya, *J. Chem. Phys*. 2023, **158**, 164103.
6. T. Kai, T. Toigawa, Y. Matsuya, Y. Hirata, T. Tezuka, H. Tsuchida and A. Yokoya, *RSC adv*. 2023, **13**, 32371–32380.
7. H. G. Paretzke, D. T. Goodhead, I. G. Kaplan and M. Terrissol, M. Track structure quantities. In: Atomic and molecular data for radiotherapy and radiation research, Chapter 9. (IAEA-TECDOC-799) IAEA, Vienna (1995).
8. M. Faubel and B. Steiner, "Photoelectron spectroscopy at liquid water surfaces," in *Linking the Gaseous and Condensed Phases of Matter: The Behavior of Slow Electrons*, NATO ASI Series B Vol. 326, edited by Christophorou, L. G., Illenberger, E., and Schmidt W. F. (Plenum Press, New York, 1994), pp. 517–523.
9. G. Moliere, *Astrophys. Phys. Phys. Chem*. 1948, **3**, 78–97.
10. J. C. Ashley, *J. Electr. Spectr. Rel. Phen.* 1990, **50**, 323-334.
11. J. M. Heller, R. N. Hammt, R. D. Birkhofft and L. R. Painter, *J. Chem. Phys.* 1974, **60**, 3483–3486.
12. H. Yada, M. Nagai and K. Tanaka, *Chem. Phys. Lett.* 2008, **464**, 166–170.
13. H. Tomita, M. Kai, T. Kusama and A. Ito, *Radiat. Environ. Biophys*. 1997, **36**, 105–116.
14. K. Takayanagi, "Introduction to electron-molecule collisions," in *Electron-Molecule Collisions*, edited by Shimamura, I. & Takayanagi, K. (Plenum Press, New York, 1984), pp. 1–87.
15. I. KrajcarBronić, M. Kimura and M. Inokuti, *J. Chem. Phys.* 1995, **102**, 6552- 6558.
16. B. Grosswendt and E. Waibel *Nucl Instr Methods* 1978, **155**, 145–156.
